# Performance of Gelatin Films Reinforced with Cloisite Na^+^ and Black Pepper Essential Oil Loaded Nanoemulsion

**DOI:** 10.3390/polym13244298

**Published:** 2021-12-09

**Authors:** Tascila F. da S. Saranti, Pamela T. S. Melo, Miguel A. Cerqueira, Fauze A. Aouada, Marcia R. de Moura

**Affiliations:** 1 Grupo de Compósitos e Nanocompósitos Híbridos (GCNH), São Paulo State University (Unesp), School of Engineering, Ilha Solteira 15385-000, SP, Brazil; tascila_tfs@hotmail.com (T.F.d.S.S.); pamelathais_@hotmail.com (P.T.S.M.); fauze.aouada@unesp.br (F.A.A.); 2 International Iberian Nanotechnology Laboratory, Av. Mestre Jose Veiga, 4715-330 Braga, Portugal; miguel.cerqueira@inl.com

**Keywords:** nanocomposite films, mechanical properties, black pepper nanoemulsion, biopolymer packaging

## Abstract

The concern about consuming eco-friendly products has motivated research in the development of new materials. Therefore, films based on natural polymers have been used to replace traditional polymers. This study consists of a production of films based on gelatin reinforced with black pepper essential oil-loaded nanoemulsions and Cloisite Na^+^. The films were characterized by water vapor permeability, mechanical and thermal properties, surface contact angle, X-ray diffraction and scanning electron microscopy. It was observed that the films containing the nanoemulsion have higher permeability values and an increase in their mechanical resistance. The addition of nanoclay contributed to an increase in the surface hydrophobicity of the film and an increase in the tensile strength, at break, by about 150%. The addition of essential oil nanoemulsions led to an increase in thermal stability. The presence of clay dispersion contributed to the formation of a surface that was slightly rougher and grainier. The addition of the black pepper essential oil nanoemulsion resulted in an increase in porosity of the gelatin matrix. Through X-ray diffraction analysis, it was possible to conclude that both the polymeric gelatin matrix and the essential oils nanoemulsion are intercalated with the clay dispersion.

## 1. Introduction

Biodegradable plastics made from renewable sources can, in some cases, replace traditional plastics derived from fossil resources (oil) [1,2,3,4]. Today, many studies have been performed with the aim of developing new biodegradable polymeric materials from renewable sources for the food industry [5,6,7,8,9]. Traditionally, the main function of food packaging is to keep the quality and safety of the food products during storage and transportation, such as extending the product shelf-life by preventing conditions and factors that are unfavorable to their conservation [10,11]. Besides the primary properties mentioned above, other functions are performed, namely: to reduce the humidity gain or loss, to prevent the contamination of microorganisms, to act as a barrier against the permeation of oxygen, carbon dioxide, and volatile compounds, as odor [10]. Thus, new technologies have been studied in order to provide safe food products, minimizing losses and waste, as well as to contribute to environmental preservation [12,13]. In addition, one of the biggest problems concerning public health is related to contamination by foodborne microorganisms. Studies have demonstrated a decrease in the proliferation of foodborne microorganisms when a package containing antimicrobial functionality was used [12,13,14,15,16]. There is a growing interest in using renewable and natural raw materials directed toward the production of films and coatings that have antimicrobial function. Plant extracts and biopolymers have been widely studied for this purpose [17,18,19]. Meanwhile, the main challenge is the development of materials that achieve an adequate balance between durability and biodegradability to ensure food product quality and low environmental impact [20].

Gelatin is a biopolymer that has the potential to satisfy many of these requirements. It is a complex polypeptide extensively used in the food, pharmaceutical, and cosmetics industry [21,22]. Gelatin films present good mechanical resistance and high elasticity. However, they exhibit high water vapor permeability rates and low humidity resistance, thus restricting their application to food products that have an elevated humidity rate [23,24]. Hence, improving the properties of these films has been attempted through nanoparticle insertion into the polymeric matrix [25]. These nanoparticles have attracted significant interest due to their ability to act as a filler on the matrix, resulting in improvements in mechanical, thermal, barrier, and other properties [26].

Some studies suggest the application of clay to polymeric films for food packaging for improving their mechanical properties and water vapor permeability [5,25]. Cloisite Na^+^ is one of most common nanomaterial used to improve these film properties. It is a hydrated mineral aluminum silicate that belongs to a class of montmorillonite. Its structure consists of an octahedral layer of alumina between two layers of tetrahedral silicate and Na^+^ [25].

To develop these materials for antimicrobial food packaging, essential oils (EO) have been broadly studied due to their antimicrobial and antioxidant properties [9,12]. The black pepper, *Piper nigrum L*., is the most common spice used around the world due to its commercial, economical, nutritional, and medical values [27,28]. There have been no publications addressing the use of the essential oil extracted from this spice in films composed of gelatin and Cloisite Na^+^.

This study adds to the current literature on gelatin composites and helps pave the way for the technical and economical feasibility of replacing non-renewable, non-biodegradable packaging materials in short-term applications by materials that are both renewable and biodegradable.

Accordingly, this study proposes the production of gelatin-based films reinforced by Cloisite Na^+^ and black pepper essential oil-loaded nanoemulsions in order to obtain a packaging material that has improved mechanical and barrier properties for food product applications.

For this, gelatin films containing black pepper essential oil and Cloisite Na^+^ were produced by the casting method and characterized by scanning electron microscopy, water vapor permeability, mechanical properties (tensile strength and elongation at break), contact angle, thermogravimetric analysis and X-ray diffraction.

## 2. Materials and Methods

### 2.1. Materials

Type B gelatin, in a colorless powder and made from an alkaline treatment, was supplied by Royal^®^. Black pepper essential oil (CAS 84929-41-9, density 0.864–0.884 g/cm^3^, free from impurities) was obtained by fruit vapor distillation according to the manufacturer Ferquima, Vargem Grande Paulista–SP. Tween^®^ 80 (CAS 900-65-6, density 1.64 g/cm^3^) was obtained from Sigma-Aldrich (Saint Louis, MO, USA), and the clay Cloisite Na^+^ was supplied by Southern Clay Products^®^.

### 2.2. Nanoemulsion Preparation

The nanoemulsions were produced by the addition of black pepper essential oil and Tween 80^®^ in distilled water. They were mixed in a homogenizer T25 Ultra-Turrax^®^ (IKA, Staufen, Germany) at different rotations and times. The following nomenclature was used for the samples: P1 (12,000 rpm and 2 min), P2 (15,000 rpm and 2 min), P3 (12,000 rpm and 5 min), and P4 (15,000 rpm and 5 min). The concentrations used were 1% (essential oil) and 0.75% (Tween 80^®^), previously tested. The nanoemulsion suspensions were diluted and characterized for their mean particle size using a Zetasizer Nano Series (Malvern Instruments Ltd. Malvern, Worcestershire, UK) equipment to perform dynamic light scattering (DLS).

### 2.3. Clay Dispersion Preparation

The clay dispersion (1% *w*/*v*) was prepared by the addition of Cloisite Na^+^ into distilled water. First, the dispersion was continuously stirred using a magnetic mixer (500 rpm) for over 30 min to achieve complete dispersion, and then it was retained to five cycles over 1 min at 50% amplitude in an ultrasound (Branson Ultrasonics Digital Sonifier 100-132-888R with Sonicator Tip 101-135-066R—Branson Ultrasonic, Mexico) in order to achieve clay exfoliation. The dispersion was characterized for the mean particle size and zeta potential through DLS by using the Zetasizer Nano Series (Malvern Instruments Ltd. Malvern, Worcestershire, UK) equipment. The cumulant mean (z-average) diameter and the polydispersity index (PdI) were used to describe droplet size and size distribution, respectively.

### 2.4. Film Preparation

The gelatin filmogenic solution was prepared by the addition of 5% (*w*/*v*) of gelatin to distilled water, which was continuously mixed in a magnetic stirrer (500 rpm) at 60 °C until the gelatin was completely dissolved. The control sample with only gelatin was named GE. The other filmogenic dispersions were named GEP1, GEP2, GEP3, GEP4, GEDA, and GEP4DA, where P1, P2, P3, and P4, represent the nanoemulsions and DA the Cloisite Na^+^. In the films containing nanoemulsion, gelatin was added to the nanoemulsion. In the films with clay dispersion and nanoemulsion, DA was added to the nanoemulsion solution containing gelatin, previously solubilized. The nanoclay dispersion was added only in the films containing nanoemulsion with smaller particle size because, in this case, films with smaller particle size had better results in terms of mechanical properties and permeability.

**Figure 1:**
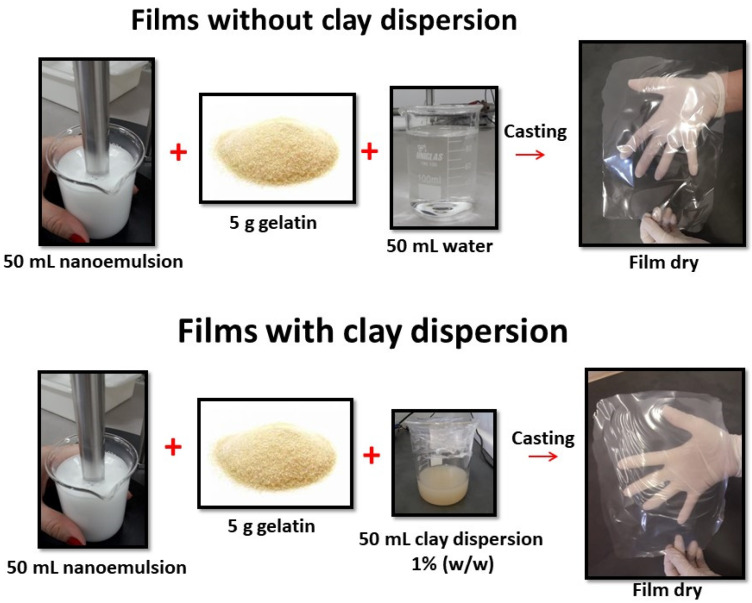
Schematic graphic of the film preparation.

The films were prepared by adding 100 mL of the filmogenic solution to a polyester support and drying for 24 h at room temperature (33 ± 5 °C) and 50 ± 2% RH. Prior to the characterization, the film samples were maintained for 48 h at 25 ± 2 °C and a constant relative humidity of 50 ± 2%. Figure 1 shows the scheme of the film preparation and the Table 1 each film composition. Afterward, the films were stored in a cabinet and the RH were controlled using silica gel and a thermo hygrometer at 25 ± 2 °C and a relative humidity of 50 ± 2% before the characterizations.

### 2.5. Film Characterization

#### 2.5.1. Scanning Electron Microscopy (SEM)

The morphology analysis of the film surface and cross section (prepared by cryogenic fractures through the deposition of samples in liquid nitrogen) were evaluated using scanning electron microscopy (SEM) EVO LS15 (ZEISS, Jena, Germany) with an operating voltage of 5 kV to 10 kV. Samples were fixed on the support using a double-sided adhesive carbon tape and sputter-coated with gold, which was carried out using the Sputter Coater Quorum, model Q150T. The gold sputtering was realized with AC current to 20 mA and deposition time equal to 1 min 30 s for each 3 nm of thickness.

#### 2.5.2. Water Vapor Permeability (WVP)

Water vapor permeability values were obtained based on the modified gravimetric method as described elsewhere [29]. The samples were shaped into circles and fixed in poly(methyl methacrylate) cells with 6 mL of deionized water and maintained at 25 ± 2 °C and relative humidity of 50 ± 2% during 25 h (monitored by a portable thermo hygrometer and maintained by periodically supplying suitable amounts of dried silica). The weight variation was determined during this period using a high precision scale. At least nine samples were performed for each film.

#### 2.5.3. Mechanical Properties

The mechanical properties were determined by using an Instron Universal Testing Machine (3369 model, Instron Corp., Canton, MA, USA) apparatus, running with a 500 N load cell and a stretch speed of 10 mm/min. To evaluate the tensile strength and elongation at break, the specimens were exposed to a tensile test to obtain their tensile versus deformation curves. To this end, the specimens were prepared according to the standard ASTM D882-97 [30]. The films were cut into rectangles 10 cm × 1.2 cm. At least nine samples were performed for each film.

#### 2.5.4. Contact Angle

The static contact angle measurements were recorded using a contact angle measurer from KSV Instruments (Helsinki, Finlandia) equipped by a digital camera CCD KSV-5000. The sessile drop method was used for to measure the contact angle. The standard liquid (Mili-Q water) droplet (7 μL) was placed on the film surface with a precision syringe and 100 images were automatically registered over 60 s. The angle formed between the drop and the surface was determined. Each film was subjected to five repetitions at 25 °C.

#### 2.5.5. Thermogravimetric Analysis (TG)

The thermogravimetric analysis was performed on a TA Instruments TGA Q-500 apparatus. Approximately 5–6 mg of the sample was measured and then subjected to a heating gradient from 20 °C to 600 °C. The heating rate was 10 °C/min and the nitrogen flow was 40 mL/min.

#### 2.5.6. X-ray Diffraction (XRD)

The X-ray diffraction technique was performed using a diffractometer XDR-6000 (Shimadzu, Japan) using the following parameters-Cu radiation source, λ = 0.154 nm; voltage = 30 kV; current = 40 mA; angular patterns between 4 and 50° with a scanning rate of 1°/min.

#### 2.5.7. Microbiological Analysis

Stock cultures of *E.* *coli* (ATCC 11229) and *S.* *aureus* (ATCC 6538) were maintained at −80 °C until grown in tubes with tryptic soy broth (TSB) (Acumedia Manufactures, Inc., Lansing, MI, USA) at 37 °C for 24 h, and then in different tubes with TSB at 37 °C for 12 h. The suspensions were streaked on solidified tryptic soy agar (TSA) (Acumedia Manufactures, Inc.) and incubated at 37 °C for 18–24 h.

Disk inhibition tests were performed according to CLSI [31]. Colonies isolated from the cultures were inoculated into 0.85% (*w*/*v*) NaCl solutions until 0.5 McFarland standard turbidity was matched (108 CFU mL^−1^). The suspensions were plated with swabs onto solidified Mueller Hinton agar (Becton, Dickinson and Co., Sparks, MD, USA). Film disks (1 cm diam) were exposed to UV light (110 V and 254 nm) for 2 min on each side before being placed at the midpoint of the inoculated Petri dishes, which were incubated at 37 °C for 16–18 h. The diameters of the inhibition zones around the films (colony-free areas) were measured to the nearest 0.01 mm with a digital caliper (Mitutoyo Corp., Kanagawa, Japan), and the growth below the film disks was visually evaluated. When inhibition zones were not detected, the compound was assumed to be inefficient in inhibiting microbial growth and the area was assigned as zero.

#### 2.5.8. Statistical Analysis

The experimental data were submitted to analysis of variance (ANOVA), and the means were compared using the Tukey test through the statistical software STATISTICA 10.0 (StatSoft, Inc., Tulsa, OK 74104, USA), with a 95% significance level.

## 3. Results and Discussion

### 3.1. Nanoemulsion Mean Particle Size

The values of the nanoemulsion mean particle size were 250 ± 8, 229 ± 9, 195 ± 7, and 181 ± 8 nm for P1, P2, P3, and P4, respectively. Thus, the direct influence of not only time but also the rotation speed on the particle size was observed. A decrease in the particle size when a lower rotation speed was used (P1 and P2) could be observed. When the rotation speed was kept constant and the time was varied, a decrease in the particle size (P2 and P4) was detected.

According to Fernadez et al. [32], the high energy provided during nanoemulsion preparation and the huge incidence of collision between particles results in the formation of the smallest droplets.

### 3.2. Clay Dispersion Mean Particle Size and Zeta Potential

The zeta potential consists of the potential difference between the ion surface tightly bonded to the particle surface and the neutral area (not charged particle) of the solution. There is a significant viscosity difference when compared to the solutions adjacent to the droplets. When the zeta potential is equal to 30 mV (module) or higher, the double layer repulsive force is stronger than the van der Waals attractive forces, so a possible flocculation is avoided [33,34]. A high value and negative zeta potential is important to the physical–chemical stabilization of the emulsion because the repulsive forces between droplets avoid flocculation [35,36].

The mean zeta potential to clay dispersion is equal to −34.8 mV ± 1.5. The high value of the zeta potential (module) indicates a high stability of the montmorillonite aqueous colloidal dispersion [37]. On the other hand, the particle size obtained was 309.6 nm ± 1.9, a value which is higher than the one obtained for the essential oil nanoemulsion particle size. Both of these results, not only for the zeta potential but also for the particle size, were similar to the results obtained by Flaker et al. [38], who studied the dispersion methods of montmorillonite to the aggregation in films based on gelatin.

### 3.3. Scanning Electron Microscopy (SEM)

**Figure 2:**
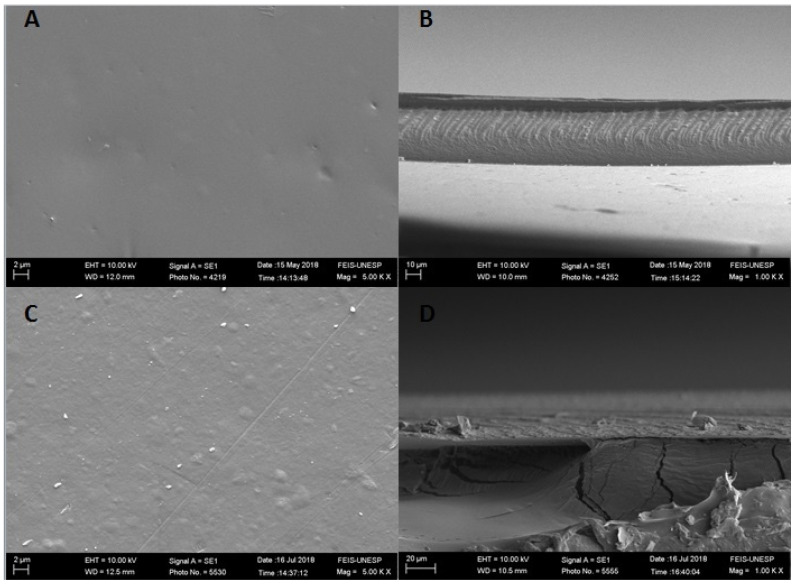
Scanning electron microscopy images of the films’ surfaces, (**A**)–GE film, (**C**)–GEDA film; cryogenic fractures, (**B**)–GE film, and (**D**)–GEDA film.

The morphology of the film surface and cross-section of the films GE and GEDA are shown in Figure 2. The image A indicates a smooth and homogeneous surface without phase separation or the formation of agglomerates.

Image C, which represents the gelatin polymeric matrix surface with the addition of clay dispersion, shows a surface that is slightly rougher and grainier, which is very similar to those presented by Alexandre et al. [25] not only for the matrix compounded only by gelation but also for the ones that contained the clay dispersion. This change on the surface of the films through the addition of clay dispersion is probably related to the agglomeration formed by the interaction between the film compounds.

**Figure 3:**
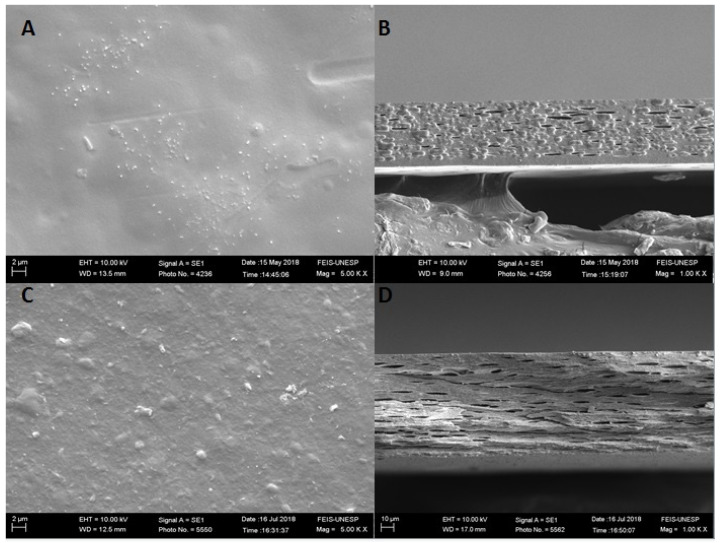
Scanning electron microscopy images of the films’ surfaces, (**A**)–GEP4 film, (**C**)–GEP4DA film; cryogenic fractures, (**B**)–GE P4 film, and (**D**)–GE P4DA film.

Figure 3 shows not only the surface morphology but also the cryogenic fractures of the GEP4 and GEP4DA films. In image A, it is possible to see a smooth, homogeneous surface without any roughness when compared to image A of Figure 2, which presents the gelatin film without the addition of the nanoemulsion. It is possible to identify that there are no changes related to the roughness of the surface. However, as can be seen in image B of Figure 3, the addition of the black pepper essential oil nanoemulsion caused changes in the microstructure of the films. It is clearly possible to verify the presence of pores in the films, indicating, according to Atarés, Bonilla and Chiralt [39], the separation of oil droplets from the aqueous phase. This occurs due to the incorporation of a compound with a hydrophobic nature into a hydrophilic phase. Possibly, the nanoemulsion addition causes a certain disturbance between the protein–protein interactions on the polymeric network, hindering the orderly alignment of the chains and, consequently, causing heterogeneity in the system, as shown in the cross-sectional micrographs.

Similar results were observed by Bonilla et al. [40] thorough the incorporation of eugenol and garlic essential oil into a gelatin and chitosan polymeric matrix and by Cordoba et al. [41], who incorporated active compounds nanoemulsion into gelatin and blends of gelatin matrix.

The clay dispersion incorporation into a matrix already containing the nanoemulsion also affects the roughness, increasing it, as observed for the control film. In the cross section image, it is possible to observe that the material porosity was maintained. Nevertheless, the oil droplets are not present in the material, and this possibly occurred due to a better interaction between the clay dispersion and the nanoemulsion; in other words, the nanostructures remained between the clay plates, preventing the oil droplets from detaching during the film drying process.

This fact is probably associated with the particle size present of nanoemulsion. The mean particle size probably causes a perturbation in the interaction between the protein chains, guiding a larger distance and, hence, larger disorder on the polymeric network.

The superficial roughness of the films containing the clay dispersion may be related to the contact angle attained at each surface; in other words, surfaces that become rougher after the addition of the clay dispersion also shows higher contact angles, and, thus, more hydrophobic surfaces. The same was observed for all film variations.

### 3.4. Water Vapor Permeabilty (WVP)

Table 2 presents the WVP obtained for the produced films. The control film, GEP4 and GEDA presented a lower value of WVP (*p* < 0.05). The addition of the essential oil and clay dispersion resulted in an increase in these values for the GEP4DA films.

The WVP values are dependent on the microstructural characteristics of the films. This increase in permeability attributed to the oil addition may be related to the appearance of a more porous and heterogeneous microstructure after it was added. This can be seen in the micrographs presented in the discussion of the scanning electron microscopy analysis. Behind the microstructure characteristics previously mentioned, the permeability can also be affected by the presence of the plasticizer. Once these materials confer more flexibility to the polymeric structure, an increase in the mobility of the dissolved water inside the matrix will result; thus, the water vapor can permeate more easily [42]. The nanoemulsion can exhibit a plasticizer effect, hindering the interaction between the gelatin chains. [43,44]. This result corroborates with those of the mechanical performance that will be discussed later, since the elongation of the films increased with the addition of oil. Similar results were obtained by Altiok et al. [45], who produced chitosan films with thyme oil. The authors also found a decrease in tensile strength and water vapor permeability after oil was added. Kavoosi et al. [46] reported a decrease in WVP after Zataria multiflora essential oil was added to gelatin films. Nunes et al. [47] also reported a decrease in water vapor permeability and tensile strength after lemon essential oil was added to gelatin matrices.

Also, the nanoemulsion particle size influences the WVP results. The film with the smallest particle size (GEP4) has the lowest WVP value among the films containing essential oil (*p* < 0.05). The nanoemulsion has a hydrophobic part and a hydrophilic part, and the hydrophilic part interacts with the gelatin matrix. This interaction is more effective when there are particles with smaller diameters because they have a reduced contact area and are better distributed throughout the film [48]. Kavoosi et al. [46] concluded that WVP values depend on the interaction between hydrophilic (gelatin) and hydrophobic (essential oil) parts that are present on the film.

Regarding the addition of clay on the films with a smallest particle size, it was possible to verify that the presence of clay causes an increase in permeation compared to the control (GE). The clay addition and the smaller particle can be causing more matrix compaction and interfering in a water vapor permeability [48].

### 3.5. Mechanical Properties

Regarding the different rotation times, it was possible to observe that this condition provoked an increase in tensile strength (*p* < 0.05), according to the Table 2 values. The smaller the particle size, the greater the observed tensile strength. By means of this result, we chose a nanoemulsion obtained through a higher rotation time—one that results in the smallest particles—to be added the clay dispersion. The results are probably due to the smallest particles having a larger surface area, thus chemical interactions with the matrix components could be favored [3,49].

The addition of the nanoemulsion causes an increase in tensile strength values when compared with GE (Table 2). As mentioned previously, the presence of hydrophobic compounds can cause an increase in the interaction force between the gelatin chains. This may explain the increase in tensile strength values when the oils were added [50].

However, the reinforcement effect of Cloisite Na^+^ is clearer when the tensile strength values were observed. The addition of clay caused a significant increase in the tension at break values of the films when compared with the control films (around 150% increase). Previous studies have showed an improvement in the mechanical properties of protein-based films containing clay as a reinforcing agent [51]. These results are attributed to the effect of a greater polymeric matrix compaction in the films with the presence of clay. Zolfi et al. [30] detected an increase in the values of maximum tension at rupture in films of soy protein isolates when they added Cloisite Na^+^ [52,53]. On another hand, when the essential oil nanoemulsion and clay dispersion were added at the same time (GEP4DA) tensile strength decreased. This result is related to the plasticizer effect, attributed to the essential oil. The hydrophobic compounds’ increase the difficulty of the interactions between molecules of the polymer and clay particles, facilitating the mobility of the chain polymers [48].

The incorporation of nanoemulsions can cause an increase in the elongation of the films (Table 2) due to the reduction in the intermolecular force between the polymer chains [54,55]. Similar results were presented by Acevedo-Fani et al. [56] when sage oil nanoemulsions were added into alginate-based edible films.

However, the addition of clay had the opposite effect; that is, it decreased the elongation of the films. This result is expected since the addition of a reinforcing agent can hinder the flow of polymer chains during the mechanical test [42].

### 3.6. Contact Angle

The pure gelatin film (GE) presented a contact angle of 72 ± 1°, and then with the addition of clay dispersion (GEDA) this value increase to 79 ± 3°. The nanoemulsion addition caused a decrease of contact angle and GEP4 exhibited an angle of 47 ± 7°. GEP4DA films show 64 ± 5° contact angle values (Table 3).

Despite the Cloisite Na^+^ conferring a hydrophilic characteristic to all films, the addition of the clay dispersion aggregated a hydrophobicity to the material surface. This fact is due to the electrostatic compatibility between the polymeric matrix and clay. The more polar is the polymeric matrix, the better is the ionic interaction between their chains and the clay structure.

However, the addition of the essential oil nanoemulsion into the polymeric matrix led to a decrease in the hydrophobic character of the surface; in other words, even though lipids originally had a hydrophobic characteristic, their addition in the form of a nanoemulsion in the polymer matrix led to a decrease in relation to their hydrophobicity. Cordoba and Sobral [41] also observed, in their study of garlic essential oil nanoemulsion in a gelatin and chitosan polymeric matrix, a decrease in relation to the contact angle after the nanoemulsion was added (*p* < 0.05).

Another point to be noted is that even with the hydrophilic nature of the gelatin, the contact angle presented by the sample composed only with gelatin (GE) indicated a hydrophobic surface; this may be due to the strong intermolecular interactions between the gelatin molecules [7].

### 3.7. Thermogravimetric Analysis (TG)

**Figure 4:**
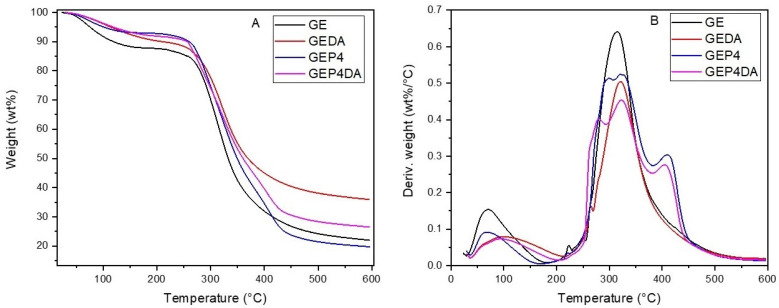
(**A**) Thermogravimetry (TG) curve of the films. (**B**) Derivative thermogravimetry (DTG) curves of the films.

The thermogravimetry (TG) and derivative thermogravimetry (DTG) curve of the films are shown in Figure 4. From the results obtained, three main peaks were identified and the initial degradation temperature (Tdi), and the final degradation temperature (Tdf) were determinate.

The pure gelatin sample (GE) had an initial degradation temperature, to the second event, at 208.84 °C and a final degradation temperature at 501.44 °C. The degradation gelatin band between 200 and 400 °C refers to the protein chain degradation [57]. For the gelatin sample incorporated by clay dispersion (GEDA), an increase in Tdi was observed this being equal to 219.56 °C. The same was observed in all samples; in other words, the clay addition showed an increase in the thermal stability of the material [53]. Similar results were obtained by Martucci, Vázquez and Ruseckaite [58]. The layered nanoclay into the polymeric matrix presented such a barrier against the heat, thus increasing the thermal stability of the material; however, in the same study, Mattucci et al. [59] observed that the addition of clay at a higher mass proportion can cause agglomeration, forming a heterogeneous dispersion in the matrix. Therefore, an increase in thermal stability was not observed.

The addition of essential oil nanoemulsions also led to an increase in thermal stability. For the sample GEP4, the second event had an initial point at 225.56 °C, in contrast to the pure gelatin polymeric matrix (GE), which was initiated at 208.84 °C. Altiok et al. [45] and Yahyaoui et al. [8] also showed that the incorporation of essential oil nanoemulsions into a polymeric matrix causes an increaase in the final thermal stability of the material.

The third event only occurred for the films with nanoemulsions in their composition, GEP4 and GEP4DA. It can be concluded that this event probably indicates the degradation of the phenolic compounds present in the oils [8].

### 3.8. X-Ray Diffraction (XDR)

The dispersion of clay platelets into the films was evaluated by the X-ray diffraction measurements. After the ultrasound treatment as previously described, the clay can be found of four different configurations, separated, intercalated, exfoliated, or intercalated/exfoliated.

**Figure 5:**
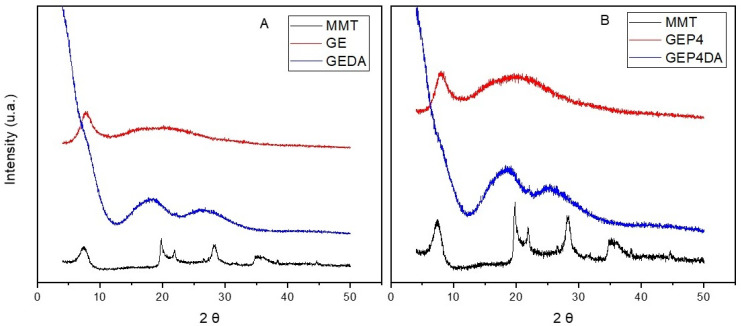
(**A**) X-ray diffractogram of MMT, GE and GEDA. (**B**) X-ray diffractogram of MMT, GEP4 and GEP4DA.

Figure 5 shows the X-ray diffractogram of the pure gelatin film (GE), the pure clay (MMT), and the gelatin film with the clay dispersion added (GEDA).

The pattern of the X-ray diffraction obtained for the pure gelatin is the typical one found in the literature. Films based on gelatin commonly show a peak at 2θ = 7°, which is attributed to the crystallinity of the triple-helix structure of gelatin, and another at 2θ = 20°, characteristic of the amorphous phase [53]. Similar results were observed by Cordoba et al. [41] and Alexandre et al. [25].

Analysis of the essential oil nanoemulsion addition into the gelatin polymeric matrix (GE–Figure 5A and GEP4–Figure 5B), a slight effect regarding the film crystallinity was observed. The same was observed by Alexandre et al. [25] when ginger essential oil was incorporated into a gelatin polymeric matrix.

It can clearly be noted from the diffractogram, through the lower diffraction peak displacement of the diffraction angles (2θ), that there was an increase in the basal spacing between the clay platelets; consequently, both the polymeric gelatin matrix and the essential oils nanoemulsion are intercalated with the clay dispersion [49].

### 3.9. Microbiological Analysis

**Figure 6:**
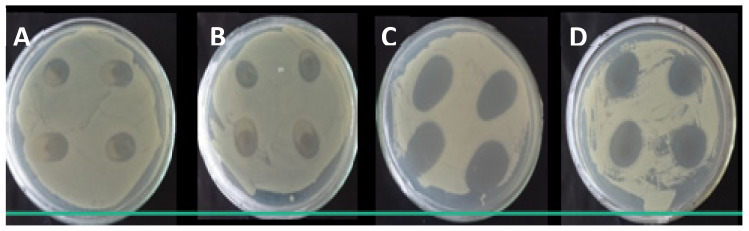
The antibacterial activity of nanocomposite by disc diffusion method: (**A**)—GE film; (**B**)—GEDA film; (**C**)—GEP4 film, and (**D**)—GEP4DA film. For each film, four samples were used to determine inhibition halo. The inhibition zone was calculated through the mean of the halo values obtained.

The antibacterial activity of nanocomposites films containing GE was determined by disc diffusion method for *E. coli* (Figure 6).

The largest inhibition halos were observed for the Gram-negative bacterium (*E. coli*). Films containing GE and Cloisite Na^+^ only, no exhibited antibacterial activity, but films containing nanoemulsion revealed by the formation of larger inhibition halos. The activation of the essential oil nanoemulsion incorporated in nanocomposite films is certain. The addition of the nanoemulsion causes an increase in inhibition halos when compared to GE and GEDA. Among the tested bacteria, the Gram-negative microorganisms *(E. coli)* were more susceptible to the action of nanoemulsion than the Gram-positive (*S. aureus*) because the active compound led to the formation of larger (*p* < 0.05) inhibition zones of the Gram-positive bacteria (Table 4).

Gram-negative bacteria have an outer hydrophilic membrane comprising lipopolysaccharide molecules that act as a bridge with hydrophilic compounds. This improves the penetration of hydrophilic compounds by nanoemulsions. Such membrane is absent in Gram-positive microorganisms, which may be explained by facilitating penetration of the microbial cell and, hence, providing the active compound with a reduced inhibitory effect. Similar observations were made by Ojagh et al. [60] and Peng and Li [61].

## 4. Conclusions

Satisfactory nanoemulsions were obtained for all rotations and times, and they were successfully incorporated into the polymeric gelatin matrix, as well as the clay dispersion, obtaining uniform, homogeneous, continuous films. It was observed that the addition of nanoemulsions increased the water vapor permeability of the films, especially in the films GEP01; this fact was observed when clay dispersion was added, increasing permeability about 20%. The black pepper was essential in increasing material deformation. On the opposite side, the clay dispersion significantly raised the tensile strength by about 150% but did not contribute to deformational change. From the thermogravimetric analysis, it was observed that the addition of the essential oil nanoemulsions and the clay dispersion promoted an increase in the thermal stability of the material. The addition of clay caused an increased contact angle in the films’ surfaces, from 72° to 79° and consequently significant hydrophobicity, allowing applications for contact with wetter products while remaining water resistant. With respect to microstructural analysis, the addition of nanoemulsions resulted in the formation of pores, and the clay dispersion caused an increase in the films’ surface roughness. Therefore, it can be said that the obtained films have great potential to be applied is active food-packaging materials.

## Figures and Tables

**Table 1:** Film composition.

Samples	Film Composition
GE	gelatin 5%
GEP1	gelatin 5% + 50 mL nanonoemulsion P1
GEP2	gelatin 5% + 50 mL nanonoemulsion P2
GEP3	gelatin 5% + 50 mL nanonoemulsion P3
GEP4	gelatin 5% + 50 mL nanonoemulsion P4
GEDA	gelatin 5% + 50 mL clay dispersion
GEP4DA	gelatin 5% + 50 mL nanonoemulsion P4 + 50 mL clay dispersion

**Table 2:** Water vapor permeability, tensile strength, and elongation of the films.

Samples	Thickness (µm)	WVP (g mm/kPa h m^2^)	Tensile Strength	Elongation (%)
GE	48 ± 1 b	0.32 ± 0.02 c	57.16 ± 3.54 e	2.96 ± 0.39 c
GEP1	47 ± 5 b	0.61 ± 0.09 a	64.05 ± 2.61 d	7.77 ± 0.91 a
GEP2	47 ± 4 b	0.57 ± 0.06 a	67.33 ± 2.06 cd	6.83 ± 0.96 a
GEP3	47 ± 4 b	0.52 ± 0.03 ab	71.04 ± 2.75 bcd	7.08 ± 0.81 a
GEP4	66 ± 5 a	0.37 ± 0.05 c	74.23 ± 1.96 bc	5.03 ± 0.75 b
GEDA	71 ± 7 a	0.38± 0.03 bc	150.72 ± 8.54 a	2.78 ± 0.42 c
GEP4DA	70 ± 7 a	0.45 ± 0.04 abc	77.32 ± 3.21 b	1.82 ± 0.31 c

a–e: Mean values ± standard deviations (*n* = 9) followed by different letters within the same column are significantly different (*p* < 0.05).

**Table 3:** Contact angle (°) of the films.

Samples	Contact Angle (°)
GE	72 ± 1° ^b^
GEP4	47 ± 7° ^d^
GEDA	79 ± 3° ^a^
GEP4DA	64 ± 5° ^c^

^a–d^: Mean values ± standard deviations (*n* = 9) followed by different letters within the same column are significantly different (*p* < 0.05).

**Table 4:** Antimicrobial effect of nanoemulsion against *Escherichia coli*, and *Staphylococcus aureus*.

Films	*E. coli* (mm^2^)	*S. aureus* (mm^2^)
GE	0	0
GEDA	0	0
GEP4	95.50 ± 0.53 ^a^	1.20 ± 0.10 ^c^
GEP4DA	49.35 ± 0.90 ^b^	0

^a–c^: Mean values ± standard deviations (*n* = 9) followed by different letters within the same column are significantly different (*p* < 0.05).

## References

[1] Bendahou D., Bendahou A., Grohens Y., Kaddami H. (2015). New nanocomposite design from zeolite and poly(lactic acid). Ind. Crops Prod..

[2] Souza V.G.L., Fernando A.L. (2016). Nanoparticles in food packaging: Biodegradability and potential migration to food—A review. Food Packag. Shelf Life.

[3] Garavand F., Cacciotti I., Vahedikia N., Rehman A., Tarhan Ö., Akbari-Alavijeh S., Shaddel R., Rashidinejad A., Nejatian M., Jafarzadeh S. (2020). A comprehensive review on the nanocomposites loaded with chitosan nanoparticles for food packaging. Crit. Rev. Food Sci. Nutr..

[4] Qin M., Chen C., Song B., Shen M., Cao W., Yang H., Zeng G., Gong J. (2021). A review of biodegradable plastics to biodegradable microplastics: Another ecological threat to soil environments?. J. Clean. Prod..

[5] Souza V.G.L., Pires J.R., Rodrigues P.F., Lopes A.A., Fernandes F.M., Duarte M.P., Fernando A.L. (2018). Bionanocomposites of chitosan/montmorillonite incorporated with *Rosmarinus officinalis* essential oil: Development and physical characterization. Food Packag. Shelf Life.

[6] Lee J.H., Lee J., Song K.B. (2015). Development of a chicken feet protein film containing essential oils. Food Hydrocoll..

[7] Shaaban H.A., Mahmoud K.F., Ibrahim M.A., Ibrahim G. (2014). Antimicrobial activity of edible methyl cellulose films enriched with essential oils against three common foodborne pathogens. World Appl. Sci. J..

[8] Yahyaoui M., Gordobil O., Díaz R.H., Abderrabba M., Labidi J. (2016). Development of novel antimicrobial films based on poly(lactic acid) and essential oils. React. Funct. Polym..

[9] Cozmuta A.M., Turila A., Apjok R., Ciocian A., Cozmuta L.M., Peter A., Nicula C., Galić N., Benković T. (2015). Preparation and characterization of improved gelatin films incorporating hemp and sage oils. Food Hydrocoll..

[10] Rhim J.-W., Park H.-M., Ha C.-S. (2013). Bio-nanocomposites for food packaging applications. Prog. Polym. Sci..

[11] Cacciotti I., Mori S., Cherubini V., Nanni F. (2018). Eco-sustainable systems based on poly(lactic acid), diatomite and coffee grounds extract for food packaging. Int. J. Biol. Macromol..

[12] Beverlya R.L., Janes M.E., Prinyawiwatkula W., No H.K. (2008). Edible chitosan films on ready-to-eat roast beef for the control of *Listeria monocytogenes*. Food Microbiol..

[13] Krepker M., Shemesh R., Poleg Y.D., Kashi Y., Vaxman A., Segal E. (2017). Active food packaging films with synergistic antimicrobial activity. Food Control.

[14] Kujumgiev A., Tsvetkova I., Serkedjieva Y., Bankova V., Christov R., Popov S. (1999). Antibacterial, antifungal and antiviral activity of propolis of different geographic origin. J. Ethnopharmacol..

[15] Muriel-Galet V., Cran M.J., Bigger S.W., Hernández-Muñoz P., Gavara R. (2015). Antioxidant and antimicrobial properties of ethylene vinyl alcohol copolymer films based on the release of oregano essential oil and green tea extract components. J. Food Eng..

[16] Gherardi R., Becerril R., Nerin C., Bosetti O. (2016). Development of a multilayer antimicrobial packaging material for tomato puree using an innovative technology. LWT.

[17] Solaberrieta I., Jiménez A., Cacciotti I., Garrigós M.C. (2020). Encapsulation of Bioactive Compounds from *Aloe Vera* Agrowastes in Electrospun Poly (Ethylene Oxide) Nanofibers. Polymers.

[18] Jovanović J., Ćirković J., Radojković A., Mutavdžić D., Tanasijević G., Joksimović K., Bakić G., Branković G., Branković Z. (2021). Chitosan and pectin-based films and coatings with active components for application in antimicrobial food packaging. Prog. Org. Coat..

[19] Burt S. (2004). Essential oils: Their antibacterial properties and potential applications in foods—A review. Int. J. Food Microbiol..

[20] Kale G., Kijchavengkul T., Auras R., Rubino M., Selke S.E., Singh S.P. (2007). Compostability of Bioplastic Packaging Materials: An Overview. Macromol. Biosci..

[21] Nitsuwat S., Zhang P., Ng K., Fang Z. (2021). Fish gelatin as an alternative to mammalian gelatin for food industry: A meta-analysis. LWT.

[22] Cetinkaya T., Altay F., Ceylan Z. (2021). A new application with characterized oil-in-water-in-oil double emulsions: Gelatin-xanthan gum complexes for the edible oil industry. LWT.

[23] Rao Y. (2007). Gelatin-clay nanocomposites of improved properties. Polymer.

[24] Suderman N., Isa M.I.N.M., Sarbon N.M. (2018). The effect of plasticizers on the functional properties of biodegradable gelatin-based film: A review. Food Biosci..

[25] Alexandre E., Lourenço R.V., Bittante A.M.Q.B., Moraes I., Sobral P.J.D.A. (2016). Gelatin-based films reinforced with montmorillonite and activated with nanoemulsion of ginger essential oil for food packaging applications. Food Packag. Shelf Life.

[26] Ray S.S., Okamoto M. (2003). Polymer/layered silicate nanocomposites: A review from preparation to processing. Prog. Polym. Sci..

[27] Sousa A.I., Ferreira I.M., Faria M.A. (2019). Sensitive detection of *Piper nigrum* L. adulterants by a novel screening approach based on qPCR. Food Chem..

[28] Nisha P., Singhal R.S., Pandit A.B. (2009). The degradation kinetics of flavor in black pepper (*Piper nigrum* L.). J. Food Eng..

[29] Espitia P.J.P., Avena-Bustillos R.J., Du W.X., Teófilo R.F., Soares N.F., McHugh T.H. (2014). Optimal antimicrobial formulation and physical-mechanical properties of edible films based on açaí and pectin for food preservation. Food Packag. Shelf Life.

[30] American Society for Testing and Materials (1996). Standard Test Method for Tensile Properties of Thin Plastic Sheeting D882-97. Annual Book of ASTM Standards.

[31] Clinical and Laboratory Standards Institute (CLSI) (2012). Performance Standards for Antimicrobial Disk Susceptibility Tests.

[32] Fernandez P., André V., Rieger J., Kühnle A. (2004). Nano-emulsion formation by emulsion phase inversion. Colloids Surf. A Physicochem. Eng. Asp..

[33] Lorevice M.V., Otoni C., de Moura M.R., Mattoso L.H.C. (2016). Chitosan nanoparticles on the improvement of thermal, barrier, and mechanical properties of high- and low-methyl pectin films. Food Hydrocoll..

[34] Calvo P., Remunan-Lopez C., Vila-Jato J.L., Alonso M.J. (1997). Novel hydrophilic chitosan-polyethylene oxide nanoparticles as protein carriers. J. Appl. Polym. Sci..

[35] Benita S., Levy M. (1993). Submicron Emulsions as Colloidal Drug Carriers for Intravenous Administration: Comprehensive Physicochemical Characterization. J. Pharm. Sci..

[36] Melo P.T.S., Aouada F.A., Moura M.R.D. (2017). Fabricação de filmes bionanocompósitos à base de pectina e polpa de cacau com potencial uso como embalagem para alimentos. Quím. Nova.

[37] Chen P., Zhang L. (2006). Interaction and Properties of Highly Exfoliated Soy Protein/Montmorillonite Nanocomposites. Biomacromolecules.

[38] Flaker C.H.C., Lourenço R.V., Bittante A.M., Sobral P.J. (2015). Gelatin-based nanocomposite films: A study on montmorillonite dispersion methods and concentration. J. Food Eng..

[39] Atarés L., Bonilla J., Chiralt A. (2010). Characterization of sodium caseinate-based edible films incorporated with cinnamon or ginger essential oils. J. Food Eng..

[40] Bonilla J., Poloni T., Lourenço R.V., Sobral P.J. (2018). Antioxidant potential of eugenol and ginger essential oils with gelatin/chitosan films. Food Biosci..

[41] Córdoba L.J.P., Sobral P.J. (2017). Physical and antioxidant properties of films based on gelatin, gelatin-chitosan or gelatin-sodium caseinate blends loaded with nanoemulsified active compounds. J. Food Eng..

[42] Cacciotti I., Lombardelli C., Benucci I., Esti M. (2019). Clay/chitosan biocomposite systems as novel green carriers for covalent immobilization of food enzymes. J. Mater. Res. Technol..

[43] Bergo P., Sobral P. (2007). Effects of plasticizer on physical properties of pigskin gelatin films. Food Hydrocoll..

[44] Ji M., Wu J., Sun X., Guo X., Zhu W., Li Q., Shi X., Tian Y., Wang S. (2021). Physical properties and bioactivities of fish gelatin films incorporated with cinnamaldehyde-loaded nanoemulsions and vitamin C. LWT.

[45] Altiok D., Altiok E., Tihminlioglu F. (2010). Physical, antibacterial and antioxidant properties of chitosan films incorporated with thyme oil for potential wound healing applications. J. Mater. Sci. Mater. Med..

[46] Kavoosi G., Rahmatollahi A., Dadfar S.M.M., Purfard A.M. (2014). Effects of essential oil on the water binding capacity, physico-mechanical properties, antioxidant and antibacterial activity of gelatin films. LWT.

[47] Nunes J.C., Melo P.T.S., Aouada F.A., Moura M.R.D. (2018). Influência da nanoemulsão de óleo essencial de limão em filmes à base de gelatina. Quím. Nova.

[48] De Moura M.R., Aouada F.A., Avena-Bustillos R.J., McHugh T.H., Krochta J.M., Mattoso L.H.C. (2009). Improved barrier and mechanical properties of novel hydroxypropyl methylcellulose edible films with chitosan/tripolyphosphate nanoparticles. J. Food Eng..

[49] Cao T.L., Song K.B. (2019). Active gum karaya/Cloisite Na^+^ nanocomposite films containing cinnamaldehyde. Food Hydrocoll..

[50] Mendes J.F., Norcino L.B., Martins H.H.A., Manrich A., Otoni C.G., Carvalho E.E.N., Piccoli R.H., Oliveira J.E., Pinheiro A.C.M., Mattoso L.H.C. (2020). Correlating emulsion characteristics with the properties of active starch films loaded with lemongrass essential oil. Food Hydrocoll..

[51] Zolfi M., Khodaiyan F., Mousavi M., Hashemi M. (2014). The improvement of characteristics of biodegradable films made from kefiran–whey protein by nanoparticle incorporation. Carbohydr. Polym..

[52] Benucci I., Liburdi K., Cacciotti I., Lombardelli C., Zappino M., Nanni F., Esti M. (2018). Chitosan/clay nanocomposite films as supports for enzyme immobilization: An innovative green approach for winemaking applications. Food Hydrocoll..

[53] Benucci I., Lombardelli C., Cacciotti I., Esti M. (2020). Papain Covalently Immobilized on Chitosan–Clay Nanocomposite Films: Application in Synthetic and Real White Wine. Nanomaterials.

[54] Vahedikia N., Garavand F., Tajeddin B., Cacciotti I., Jafari S.M., Omidi T., Zahedi Z. (2019). Biodegradable zein film composites reinforced with chitosan nanoparticles and cinnamon essential oil: Physical, mechanical, structural and antimicrobial attributes. Colloids Surf. B Biointerfaces.

[55] Otoni C.G., de Moura M.R., Aouada M.R.D.M., Camilloto G.P., Cruz R.S., Lorevice M.V., Soares N.D.F., Mattoso L.H. (2014). Antimicrobial and physical-mechanical properties of pectin/papaya puree/cinnamaldehyde nanoemulsion edible composite films. Food Hydrocoll..

[56] Acevedo-Fani A., Salvia-Trujillo L., Rojas-Graü M.A., Martín-Belloso O. (2015). Edible films from essential-oil-loaded nanoemulsions: Physicochemical characterization and antimicrobial properties. Food Hydrocoll..

[57] Roy S., Rhim J.W. (2021). Gelatin/agar-based functional film integrated with Pickering emulsion of clove essential oil stabilized with nanocellulose for active packaging applications. Colloids Surf. A Physicochem. Eng. Asp..

[58] Martucci J.F., Vazquez A., Ruseckaite R.A. (2007). Nanocomposites based on gelatin and montmorillonite. J. Therm. Anal. Calorim..

[59] Pereda M., Ponce A.G., Marcovich N.E., Ruseckaite R.A., Martucci J.F. (2011). Chitosan-gelatin composites and bi-layer films with potential antimicrobial activity. Food Hydrocoll..

[60] Ojagh S.M., Rezaei M., Razavi S.H., Hosseini S.M.H. (2010). Development and evaluation of a novel biodegradable film made from chitosan and cinnamon essential oil with low affinity toward water. Food Chem..

[61] Peng Y., Li Y. (2014). Combined effects of two kinds of essential oils on physical, mechanical and structural properties of chitosan films. Food Hydrocoll..

